# A New Comma Bacillus

**Published:** 1885-09

**Authors:** W. D. Miller

**Affiliations:** Berlin


### A NEW COMMA BACILLUS.

BY DR. W. D. MILLER.

Considering the great interest which for some months has centered around the so-called comma bacilli, a short description of a new member of this probably very numerous and widely distributed family may not be out of place.

In the month of May, I had occasion to spend a day in Egerstorff, near Hanover, and in the gutter water, as also in the drinking water of this untidy village, I could easily detect, by the use of the microscope, the presence of a comma bacillus. Its pure culture was accomplished on agar-agar without much difficulty; on this medium it grows rapidly at blood or room temperature, and forms on the surface a thin, bluish, semi-liquid layer, one-half to one mm. thick. On ten per cent. peptone-gelatine it grows rapidly at first, but after forty-eight hours its growth seems to cease altogether. On plates, in the second or third dilution, after twenty-four hours' growth, the colonies appear, under one hundred diameters, very irregular, lobular, grayish in color, and traversed by fissures. It differs from all comma bacilli yet discovered, in that it liquefies neither gelatine nor blood-serum. On potato it grows poorly at all temperatures. It is somewhat smaller than the comma bacillus which I isolated from the oral secretions, and possesses in a high degree the peculiarity characteristic of the other comma bacilli, of assuming a great variety of shapes and sizes. In growing colonies on agaragar, the cells are tolerably regular, comma or S-shaped; occasionally one finds spirilla, or long, closely twisted leptothrix. In older cultures, on the other hand, the cells become mostly round, or oval, or lemon-shaped, sometimes much increased in size, while the protoplasm is granularly differentiated. In gelatine the cells are mostly short and plump, while on potato almost any form may be found.

The statement of Klein, that comma bacilli occur in the cœcum of healthy guinea-pigs, I have been able to confirm. I did not, however, find them in such numbers as I had expected, after reading his report. Attempts at pure culture did not succeed, so that it would be hazardous in the extreme to make any suppositions as to the identity of these guinea-pig bacilli with any of the known comma bacilli.

BERLIN, June 12, 1885.

